# Complete Response in Recurrent End-Stage Pancreatic Cancer After Combined Immune Cell Therapy of α-Galactosylceramide Dendritic Cell Vaccine Therapy, Wilms’ Tumor 1 Dendritic Cell Vaccine and Natural Killer Cell Therapy

**DOI:** 10.7759/cureus.83607

**Published:** 2025-05-06

**Authors:** Keibun Liu, Hisashi Nagai, Nobuo Kanai, Kenichi Yamahara

**Affiliations:** 1 Oncology, Ginza Phoenix Clinic, Tokyo, JPN; 2 Human and Environmental Studies, Tokai University, Hiratsuka, JPN; 3 Healthy Aging Innovation Center, Tokyo Metropolitan Institute for Geriatrics and Gerontology, Tokyo, JPN; 4 Institute for Advanced Medical Sciences, Hyogo Medical University, Nishinomiya, JPN

**Keywords:** dendritic cell, natural killer cell, pancreatic cancer, wilms' tumor antigen 1, α-galactosylceramide

## Abstract

Pancreatic cancer exhibits high mortality and morbidity, and the prognosis worsens when recurrence occurs. Current standard treatments, such as chemotherapy or radiation, show limited efficacy in recurrent end-stage pancreatic cancer. A man in his 70s visited our facility with a diagnosis of recurrent end-stage pancreatic cancer (Stage Ⅳ) with multiple metastases to mesenteric and paratracheal lymph nodes and the right liver lobe. Due to the severe side effects, chemotherapy was not an option anymore. After apheresis, a combined immune cell-based therapy incorporating α-galactosylceramide (α-Galcer)-pulsed dendritic cell (DC) vaccine therapy into Wilms' tumor 1 (WT-1) vaccine and natural killer (NK) cell therapy was initiated. On completing a nearly three-month course of seven administrations of each therapy, follow-up positron emission tomography-computed tomography (PET-CT) scans showed the regression of the recurrent cancer region as well as the metastasis regions. Meanwhile, chemotherapy was not provided. A significant decrease in tumor-associated markers (i.e., CA19-9, from 88.3 to 54.4 U/mL; DU-PAN-2, from 267 to 76 U/mL) and improvement in the immune profile status (i.e., neutrophil percentage decreased from 68% to 64.2%; lymphocyte percentage increased from 18% to 25.6%, and neutrophil-lymphocyte ratio decreased from 3.8 to 2.5) were observed. His performance status initially deteriorated (to 1 or 2), but improved to 0 without any disability and limiting daily life. Any adverse events or side effects associated with the immune cell-based therapy were not recorded. This single-patient case report demonstrated the clinical potential of incorporating α-Galcer DC vaccine therapy into WT-1 vaccine and NK cell therapy to enhance the anti-tumor effects of immune cell-based therapy. While this case report provided significant insights to facilitate future research, further observations and investigations are warranted with the ultimate goal of improving outcomes in patients with advanced cancers.

## Introduction

Pancreatic cancer remains one of the most lethal malignancies worldwide, characterized by its aggressive nature, late diagnosis, and limited therapeutic options [1]. Despite advancements in surgical techniques, chemotherapy regimens, and radiation options, the overall five-year survival rate of patients diagnosed with advanced pancreatic cancer has been reported to be only 2% [2,3]. Even among patients who undergo curative resection, the recurrence rate is as high as nearly 80%, most commonly within the first two years after surgery [4]. Recurrent pancreatic cancer poses an even greater therapeutic challenge, largely due to the limited efficacy and applicability of standard treatments, declining performance status, or resistance developed during prior treatment regimens [5,6]. It is significantly associated with poor survival outcomes; the median overall survival after recurrence is typically 6 to 11 months, depending on the site and timing of recurrence as well as the patient’s performance status and available treatment options [7].

These situations have fueled a growing interest in alternative therapeutic strategies, particularly immunotherapy [8]. Among them, autologous immune cell-based approaches have emerged as promising strategies that leverage the body's innate and adaptive immune systems to combat cancer. We previously reported that Wilms' tumor 1 (WT-1) dendritic cell (DC) vaccine therapy functions by presenting the tumor-associated antigens to T cells, thereby triggering a robust and specific cytotoxic immune response against cancer cells [9]. In addition, combining two cell therapies of WT-1 DC vaccine and natural killer (NK) cell could boost their anti-tumor effects through multiple-interactive activations even in late-stage cancer with multiple metastases [10]. However, advanced pancreatic cancer was reported to be less responsive to the immunotherapy using WT-1 DC vaccine, represented by small delayed-type hypersensitivity (DTH) and low maximum temperature after the initiation of the immune cell therapy [11]. It was reported that immunotherapy showed less responsiveness and effectiveness against pancreatic cancer due to peculiar features of the immunosuppressive tumor microenvironment. In this context, incorporating α-galactosylceramide (α-Galcer)-primed DC therapy proposes a potential next step to increase synergistic effects in enhancing antitumor immunity [12]. It offers a novel mechanism, bypassing resistance to standard chemotherapy, through activating endogenous natural killer T (NKT) cells and NKT cell-dependent responses, increasing interferon gamma (IFN-γ)-producing cells, and leading to improved immune surveillance and potential tumor control [13]. A recent phase Ⅱ clinical trial showed its benefits in prolonging the overall survival [14]. This therapy could be particularly advantageous in the recurrent setting, where conventional treatments are no longer viable.

Here, we report an insightful case of complete response in a patient with recurrent end-stage pancreatic cancer who received a combined immune cell therapy that incorporated α-Galcer-primed DC therapy into WT-1 DC and NK cell therapy, without the use of chemotherapy.

## Case presentation

**Figure 1:**
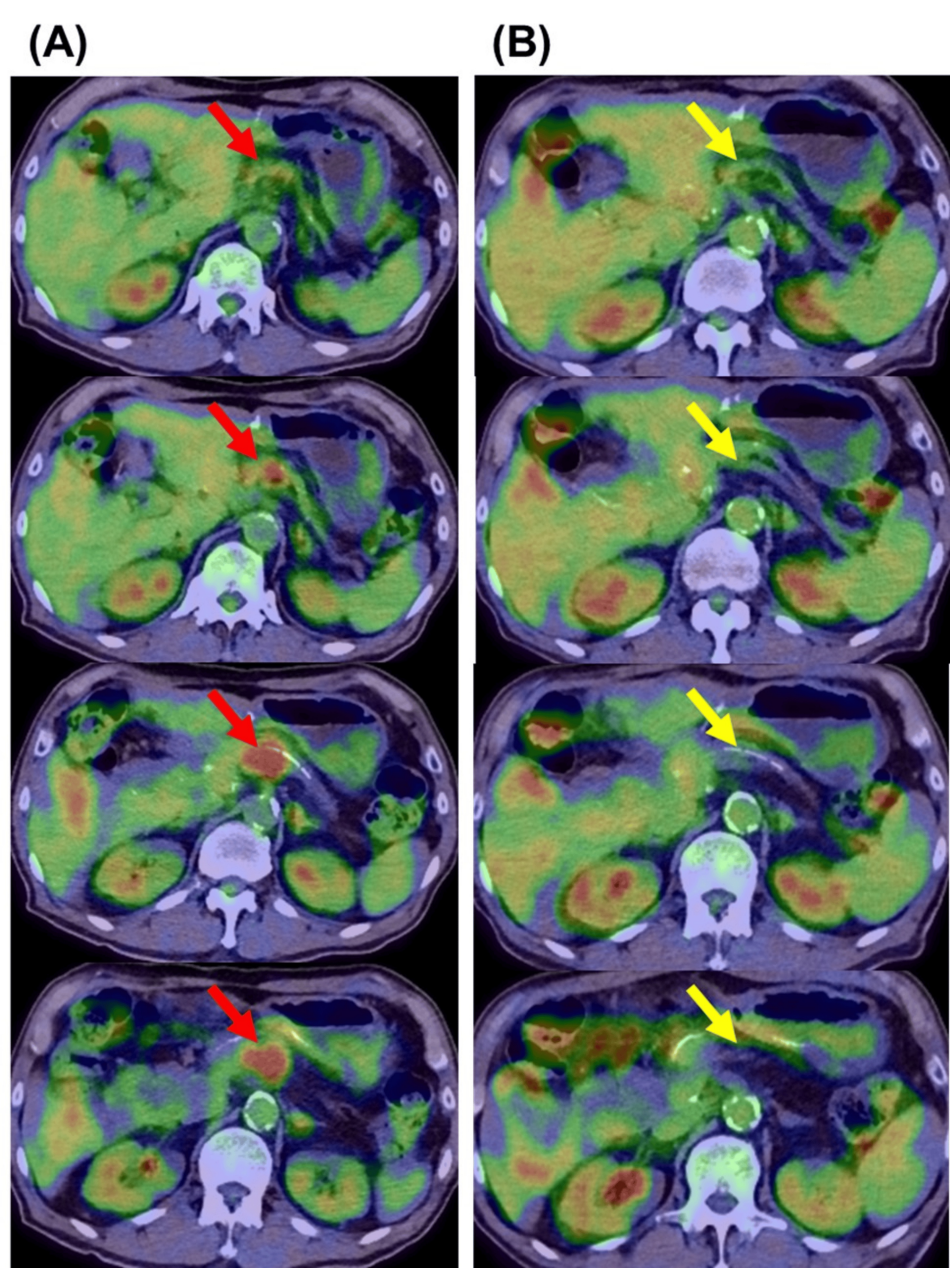
Positron emission tomography-computed tomography (PET-CT) examinations (A) before and (B) after the combined immune therapy Red arrows demonstrate the region of recurrent pancreatic cancer identified by PET-CT, and yellow arrows, performed after the immune cell therapy, demonstrate where the region of recurrent pancreatic cancer existed. The adjacent left and right images show the slices at the same layer.

A man in his 70s visited our facility with a diagnosis of recurrent end-stage pancreatic cancer (Stage Ⅳ) with multiple metastases at mesenteric and paratracheal lymph nodes and the right liver lobe. He was originally diagnosed with pancreatic head invasive ductal adenocarcinoma (Stage ⅠA) and underwent a pancreaticoduodenectomy two years ago. Local recurrent pancreatic cancer at the pancreatic cut end margin with a diameter of 28 mm (Figure 1A) was identified by positron emission tomography-computed tomography (PET-CT) one year ago at the outpatient clinic follow-up.

Despite a six-course regimen of FOLFIRINOX chemotherapy following which the size of the recurrent pancreatic tumor slightly decreased, the tumor-associated markers remained high, and new metastases were identified in the following CT examinations: metastatic mesenteric adenopathy (up to 16 mm), metastatic paratracheal adenopathy (up to 8 mm), and liver metastasis to the right lobe (up to 10 mm). These metastases were not detected in the PET examination before the FOLFIRINOX. At the time of visiting our facility, he had mildly impaired performance status and symptoms of chronic fatigue, loss of appetite and body weight, and mild abdominal distension. Due to the severe side effects of FOLFIRINOX, he could not continue chemotherapy. The laboratory investigations showed a poor immune profile status (IPS) with a high neutrophil percentage (68%), low lymphocyte percentage (18%), and an elevated neutrophil-lymphocyte ratio (3.8), anemia, impaired liver function, high biliary-associated markers, mild kidney injury, an increase in C-reactive protein and ferritin levels, indicating a chronic inflammation, and a high level of tumor-associated markers (Table 1).

**Table 1:** Characteristics of the patient CEA: carcinoembryonic antigen, CA19-9: carbohydrate antigen 19-9, DU-PAN-2: duodenal pancreatic cancer antigen 2

Variable	Before the immune therapy	After the immune therapy	Reference
Immune profile status			
White blood cell (/μL)	8400	7000	3500-9700
Neutrophil (%)	68	64.2	42-74
Lymphocyte (%)	18	25.6	18-50
Neutrophil/lymphocyte ratio	3.8	2.5	NA
Other laboratory results			
Hemoglobin (g/dL)	10.3	9.9	13.6-18.3
Hematocrit (%)	32.6	30.1	40.4-51.9
Platelets (×10^4^/μL)	16.2	15.2	14.0-37.9
Albumin (g/dL)	3.3	3.5	3.8-5.2
Total bilirubin (mg/dL)	0.4	0.4	0.3-1.2
Aspartate aminotransferase (AST) (U/L)	108	28	10-40
Alanine aminotransferase (ALT) (U/L)	164	25	5-45
Lactate dehydrogenase (LDH) (U/L)	214	194	120-245
Alkaline phosphatase (ALP) (U/L)	357	140	38-113
γ-Glutamyl transpeptidase (γGTP) (U/L)	463	65	<79
Creatinine (mg/dL)	1.03	1	0.65-1.09
Sodium (mEq/L)	140	140	135-145
Potassium (mEq/L)	4	3.7	3.5-5.0
Chloride (mEq/L)	106	105	98-108
Chronic inflammation			
C-reactive protein (CRP) (mg/dL)	0.62	0.32	<0.03
Ferritin (ng/mL)	219.4	41.7	21-282
Tumor-associated markers			
CEA (ng/mL)	4.7	3.9	<5.0
CA19-9 (U/mL)	88.3	54.4	<37.0
DU-PAN-2 (U/mL)	267	76	<150
SPan-1 (U/mL)	57.9	29.2	<30.0

**Figure 2:**
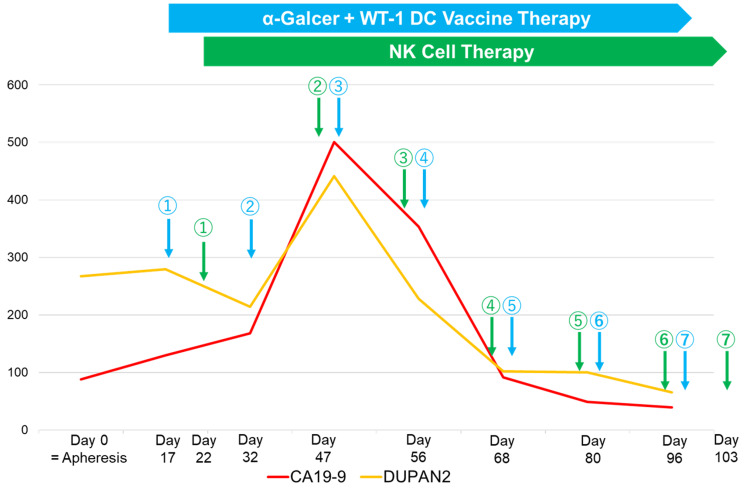
Personalized plan of a combined immune cell therapy WT1: Wilms' tumor 1, DC: dendritic cell, α-Galcer: α-galactosylceramide, NK cell: natural killer cell The numbers above the arrows indicate the order of administration.

Following apheresis, a personalized combined immune cell therapy incorporating α-Galcer DC therapy into WT-1 DC vaccine and NK cell therapy was administered (Figure 2).

The α-Galcer and WT-1 DC vaccine therapy was administered on the same day along with a small amount of nivolumab (20 mg; Ono Pharmaceutical Co., Ltd., Osaka). A 0.1-mL dose of the WT-1 DC vaccine was subcutaneously injected into the forearm to measure the DTH at every WT-1 DC administration, showing a mean of 30.0 mm (standard deviation, SD: 1.7 mm) across the seven administrations. The maximum temperature recorded in response to each administration of DC vaccine therapy had a mean of 37.9 ℃ (SD: 0.8). A significant decrease in tumor-associated markers was observed after nearly three months of the immune cell therapy (Figure 2). Meanwhile, no chemotherapy regimens were provided. The follow-up PET-CT showed the regression of the recurrent pancreatic cancer region (Figure 1B) and could not detect fluorodeoxyglucose (FDG) uptake at mesenteric, paratracheal, and liver metastases that were detected in the past CT examinations. The laboratory results showed an improvement in IPS (i.e., a decrease in the neutrophil percentage and neutrophil-lymphocyte ratio, and an increase in the lymphocyte percentage), liver function, high biliary-associated markers, and chronic inflammation (Table 1). The patient demonstrated a good performance status (0) with no symptoms and disabilities and gained weight after recovering appetite. There were no adverse events or side effects associated with the immune cell therapy, including infections, allergy, vomiting, or other symptoms conventionally seen in chemotherapy. One year following the diagnosis of recurrent pancreatic cancer, the patient still showed a good performance status.

## Discussion

This single-patient case report indicated that a combined immune cell therapy, incorporating α-Galcer DC vaccine therapy into WT-1 DC vaccine and NK cell therapy, could play an important role in enhancing the anti-tumor effects of immunotherapy. Surprisingly, the recurrent end-stage pancreatic cancer and metastatic regions were regressed, leading to a complete response, only by a combined immune cell therapy without any potential chemotherapies.

The advanced pancreatic cancer is well known as a “cold tumor”, representing remarkably low responsiveness to any therapeutic options; therefore, its prognosis (i.e., survival rate) is extremely poor [4,6,15]. Especially in recurrent end-stage pancreatic cancer, chemotherapy is the only therapeutic option, though with limited effects seen. Immune checkpoint inhibitors, which are often selected even for advanced cancer at Stage Ⅳ (i.e., lung cancer), are also ineffective in most cases and not usually used for advanced end-stage pancreatic cancer [8,16].

Recently, in this context, autologous immune cell-based therapy in the treatment of advanced cancer has drawn interest, as this could approach the pathways where the standard therapy cannot contribute. Our group reported advanced cancer cases that led to a good prognosis with the use of the immune cell-based therapy [9,10,12]. However, the responses varied among cases. We reported that the advanced pancreatic cancer showed less responsiveness to the immune cell-based therapy, represented by smaller DTH and lower maximum temperature after DC administration, and a poor prognosis compared to the advanced lung cancer [11]. In this case, the immune cell-based therapy that incorporated α-Galcer DC therapy showed a relatively high maximum temperature compared to the previous report with WT-1 DC vaccine therapy alone, indicating that α-Galcer DC therapy could improve immune responsiveness and its anti-tumor effect as well. This is probably because of the ability of DCs pulsed with α-Galcer to orchestrate and coordinate both innate and adaptive immune responses, balancing and strengthening the overall anti-tumor immunity. DCs pulsed with α-Galcer can stimulate anti-tumor responses by activating NKT cells and CD8+ T cells. Activated NKT cells play a central role in specific tumor immunology, stimulating interferon gamma (IFN-γ) production and cytokine secretion, which contribute to the enhancement of the overall T cell activation and anti-tumor functions [8,13,17]. IFN-γ or other cytokines produced via this pathway can also activate the other immune cells, stepping up multiple layers of anti-immunity mechanisms.

WT-1 ranked first among 75 common antigens in advanced cancers according to the National Cancer Institute, and has been frequently used as the target antigen for DC vaccine therapy [18]. However, its clinical efficacy as monotherapy has been limited, especially in the treatment of advanced end-stage pancreatic cancer [11,19,20]. The potential reason is that WT-1 DC therapy is too specific to the target tumor antigen (WT-1), which could also be heterogeneously expressed even in the same cancer body, potentially limiting its overall effect. This might also indicate that WT-1 DC vaccine as a monotherapy is insufficient to break the strong barrier in the tumor microenvironment developed by advanced pancreatic cancer cells, which provides protection from the anti-tumor immunity in a varied ways (i.e., presenting proteins that reduce the ability of immune cells, secreting cytokines and chemokines that promote tumor growth, using suppressive mechanisms like CTLA-4 and PD-1 to inhibit T cell functions, tumor-associated macrophages that promote angiogenesis, fibrous stroma deposition, and metastasis, etc.) [21]. As evidence of less responsibility, the DTH (a median of 30 mm in this case), an index of the immune response of WT-1 antigen-specific cytotoxic T cells, was relatively smaller than the strong response (55-90 mm) observed in a group with better outcomes in previous reports [11].

NK therapy contributes to the improved and sustained immune function by non-specifically targeting stressed cells without prior sensitization [22,23]. However, due to its non-specific function, the overall anti-tumor response as a monotherapy is also limited. In this context, the incorporation of α-Galcer-primed DC therapy may overcome limitations of both WT1 DC and NK therapies, enhancing anti-tumor responses.

As evidence of the ability of α-Galcer-primed DC therapy, the immune profile status, an indicator of the balance between neutrophils and lymphocytes, or the anti-tumor environment [9], significantly improved after the completion of the immune therapy (Table 1). An improvement in the liver function was also observed, likely due to the regression of liver metastasis as shown in the follow-up PET. Interestingly, the biliary-associated markers also decreased over time, potentially indicating the regression of micro-metastasis in the biliary duct or reduction in the obstruction in the duct induced by micro-metastasis or the recurrent cancer body itself.

In this case, we also added a small amount of an immune checkpoint inhibitor (a 20 mg dose of nivolumab) intravenously. Although immune checkpoint inhibitors are often ineffective in advanced end-stage pancreatic cancer [8,16], after breaking the tumor-associated microenvironmental barrier by the combined immune cell-based therapy, an immune checkpoint inhibitor could efficiently activate the anti-tumor effects of T or NK cells through inhibiting PD-1 binding to PD-L1 and enhancing the antibody-dependent cytotoxic activity [24]. The small amount of the immune checkpoint inhibitor was provided based on our previous report [10]. Therefore, the appropriate dosing of immune checkpoint inhibitors to combine with the immune cell therapy also requires further investigations.

We acknowledge several limitations in this case-report-based research. First, the high cost associated with the immune cell therapy limits clinical availability and its widespread usage. Second, there is no standard protocol or consensus on the dose, frequency, and interval period between administrations. Third, the incubation condition could be varied every time, depending on the patient's condition; hence, the effects of the immune cell therapy may not be consistent. Fourth, whether a combined immune cell therapy can be applied to other types of cancer warrants further investigation. Fifth, without control, we cannot conclude its causality. However, the favorable results in a patient with end-stage pancreatic cancer without any chemotherapeutic or other adjunctive options could show the potential for future investigations.

## Conclusions

We reported the complete response of recurrent end-stage pancreatic cancer after introducing a combined immune cell therapy of α-Galcer DC vaccine therapy, WT-1 DC vaccine, and NK cell therapy, without any chemotherapy or adjunctive options. Although a single-patient-based report cannot establish causality, this case facilitates further investigation of the anti-tumor effects of a combined immune cell therapy, which could offer additional options in the treatment of advanced cancers.

## References

[1] Siegel RL, Miller KD, Wagle NS, Jemal A (2023). Cancer statistics, 2023. CA Cancer J Clin.

[2] Rahib L, Smith BD, Aizenberg R, Rosenzweig AB, Fleshman JM, Matrisian LM (2014). Projecting cancer incidence and deaths to 2030: the unexpected burden of thyroid, liver, and pancreas cancers in the United States. Cancer Res.

[3] Johnson BA III, Yarchoan M, Lee V, Laheru DA, Jaffee EM (2017). Strategies for increasing pancreatic tumor immunogenicity. Clin Cancer Res.

[4] Kamisawa T, Wood LD, Itoi T, Takaori K (2016). Pancreatic cancer. Lancet.

[5] Liu B, Zhou H, Tan L, Siu KT, Guan XY (2024). Exploring treatment options in cancer: tumor treatment strategies. Signal Transduct Target Ther.

[6] Park W, Chawla A, O'Reilly EM (2021). Pancreatic cancer: a review. JAMA.

[7] Groot VP, Rezaee N, Wu W (2018). Patterns, timing, and predictors of recurrence following pancreatectomy for pancreatic ductal adenocarcinoma. Ann Surg.

[8] Waldman AD, Fritz JM, Lenardo MJ (2020). A guide to cancer immunotherapy: from T cell basic science to clinical practice. Nat Rev Immunol.

[9] Nagai H, Karube R (2023). WT1 dendritic cell vaccine therapy improves immune profile and prolongs progression-free survival in end-stage lung cancer. Cureus.

[10] Nagai H, Karube R (2024). Late-stage ovarian cancer with systemic multiple metastases shows marked shrinkage using a combination of Wilms’ tumor antigen 1 (WT1) dendritic cell vaccine, natural killer (NK) cell therapy, and nivolumab. Cureus.

[11] Nagai H, Karube R (2023). Delayed-type hypersensitivity: an excellent indicator of anti-tumor immunity with Wilms’ tumor 1 (WT1) dendritic cell vaccine therapy. Cureus.

[12] Nagai H, Chen H, Karube R, Koitabashi Y, Numata O, Yamahara K (2024). Combination of radiation therapy, Wim’s tumor 1 (WT1) dendritic cell vaccine therapy, and α-galactosylceramide-pulsed dendritic cell vaccine therapy for end-stage small bowel cancer. Cureus.

[13] Zhang Y, Springfield R, Chen S (2019). α-GalCer and iNKT cell-based cancer immunotherapy: realizing the therapeutic potentials. Front Immunol.

[14] Toyoda T, Kamata T, Tanaka K (2020). Phase II study of α-galactosylceramide-pulsed antigen-presenting cells in patients with advanced or recurrent non-small cell lung cancer. J Immunother Cancer.

[15] Ullman NA, Burchard PR, Dunne RF, Linehan DC (2022). Immunologic strategies in pancreatic cancer: making cold tumors hot. J Clin Oncol.

[16] Li X, Gulati M, Larson AC, Solheim JC, Jain M, Kumar S, Batra SK (2022). Immune checkpoint blockade in pancreatic cancer: trudging through the immune desert. Semin Cancer Biol.

[17] Matsuyoshi H, Hirata S, Yoshitake Y (2005). Therapeutic effect of alpha-galactosylceramide-loaded dendritic cells genetically engineered to express SLC/CCL21 along with tumor antigen against peritoneally disseminated tumor cells. Cancer Sci.

[18] Cheever MA, Allison JP, Ferris AS (2009). The prioritization of cancer antigens: a National Cancer Institute pilot project for the acceleration of translational research. Clin Cancer Res.

[19] Kalinski P, Muthuswamy R, Urban J (2013). Dendritic cells in cancer immunotherapy: vaccines and combination immunotherapies. Expert Rev Vaccines.

[20] Nava S, Lisini D, Frigerio S, Bersano A (2021). Dendritic cells and cancer immunotherapy: the adjuvant effect. Int J Mol Sci.

[21] Hartupee C, Nagalo BM, Chabu CY, Tesfay MZ, Coleman-Barnett J, West JT, Moaven O (2024). Pancreatic cancer tumor microenvironment is a major therapeutic barrier and target. Front Immunol.

[22] Yoon SR, Kim TD, Choi I (2015). Understanding of molecular mechanisms in natural killer cell therapy. Exp Mol Med.

[23] Sakamoto N, Ishikawa T, Kokura S (2015). Phase I clinical trial of autologous NK cell therapy using novel expansion method in patients with advanced digestive cancer. J Transl Med.

[24] Oyer JL, Gitto SB, Altomare DA, Copik AJ (2018). PD-L1 blockade enhances anti-tumor efficacy of NK cells. Oncoimmunology.

